# Experimental and Theoretical Screening for Green Solvents Improving Sulfamethizole Solubility

**DOI:** 10.3390/ma14205915

**Published:** 2021-10-09

**Authors:** Piotr Cysewski, Maciej Przybyłek, Rafal Rozalski

**Affiliations:** 1 Department of Physical Chemistry, Faculty of Pharmacy, Collegium Medicum in Bydgoszcz, Nicolaus Copernicus University in Toruń, Kurpińskiego 5, 85-950 Bydgoszcz, Poland; m.przybylek@cm.umk.pl; 2 Department of Clinical Biochemistry, Faculty of Pharmacy, Collegium Medicum in Bydgoszcz, Nicolaus Copernicus University in Toruń, Karłowicza 24, 85-950 Bydgoszcz, Poland; rafalr@cm.umk.pl

**Keywords:** sulfamethizole, solubility, machine learning, ensemble of neural networks, COSMO-RS, binary solvents, sigma potentials, green solvents

## Abstract

Solubility enhancement of poorly soluble active pharmaceutical ingredients is of crucial importance for drug development and processing. Extensive experimental screening is limited due to the vast number of potential solvent combinations. Hence, theoretical models can offer valuable hints for guiding experiments aimed at providing solubility data. In this paper, we explore the possibility of applying quantum-chemistry-derived molecular descriptors, adequate for development of an ensemble of neural networks model (ENNM), for solubility computations of sulfamethizole (SMT) in neat and aqueous binary solvent mixtures. The machine learning procedure utilized information encoded in *σ*-potential profiles computed using the COSMO-RS approach. The resulting nonlinear model is accurate in backcomputing SMT solubility and allowed for extensive screening of green solvents. Since the experimental characteristics of SMT solubility are limited, the data pool was extended by new solubility measurements in water, five neat organic solvents (acetonitrile, N,N-dimethylformamide, dimethyl sulfoxide, 1,4-dioxane, and methanol), and their aqueous binary mixtures at 298.15, 303.15, 308.15, and 313.15 K. Experimentally determined order of decreasing SMT solubility in neat solvents is the following: N,N-dimethylformamide > dimethyl sulfoxide > methanol > acetonitrile > 1,4dioxane >> water, in all studied temperatures. Similar trends are observed for aqueous binary mixtures. Since N,N-dimethylformamide is not considered as a green solvent, the more acceptable replacers were searched for using the developed model. This step led to the conclusion that 4-formylmorpholine is a real alternative to N,N-dimethylformamide, fulfilling all requirements of both high dissolution potential and environmental friendliness.

## 1. Introduction

Sulfamethizole (SMT, CAS: 144-82-1, DrugBank: DB00576) is a sulfonamide antibiotic drug that is mainly used for urinary infection treatment. Its bacteriostatic activity is typical for sulfonamides and is closely associated with the inhibition of dihydropteroate synthetase, which impedes binding of p-aminobenzoic acid (PABA) and the synthesis of folic acid involved in bacteria multiplication process. Sulfamethizole is characterized by quite low aqueous solubility (1050 mg/L at 310.15 K) [1], which is why various formulations were proposed for improving SMT bioavailability and its dissolution properties. For example, new formulations were prepared via cocrystallization [2,3,4], complexation with cyclodextrins [5], solid dispersions [6], and nanoparticles [7]. However, in some cases, the solubility must be reduced. Therefore, by optimizing binary mixture composition, one can obtain the solvent with precise characteristics suitable for a particular technological application. This includes antisolvent crystallization techniques, which have been used to obtain a formulation with the appropriate particle size characterized by improved bioavailability [8,9,10]. Multicomponent solvents have also been applied in liquid drug formulations. Water–organic solvent mixtures deserve particular attention due to frequent cosolvation and synergistic effects. The latter is characterized by a nonadditivity of solute–solvent affinities resulting in an increase of solubility in binary mixture at a certain composition compared to pure solvents. This behavior is quite common, and it is manifested by the appearance of a maximum on the molar fraction solubility plotted as a function of binary solvent composition. Some recent examples reported in the literature of such behavior include aqueous binary mixtures of nicotinamide in dimethyl sulfoxide (DMSO) [11], theophylline in 1-butanol [12], phenacetin in 1,4-dioxane [13,14], sulfanilamide in 1,4-dioxane [15], paracetamol in ethanol and propylene glycol [16], 4-(hydroxymethyl)benzoic acid in ethanol [17], and piroxicam in ethanol [18].

It should be noted that solubility enhancement is not the only criterion for solvent utilization since potential toxicity is another key factor restricting their utilization in pharmaceutical and chemical industry. Hence, screening of efficient solubilizers should adhere to the sustainable chemistry concept and ought to be as environmentally neutral as possible. For this reason, variety of solvent selection strategies are used for an assessment of a wide range of hazards including aquatic, air, persistency, irritation, chronic and acute toxicity, flammability, reactivity, and release potential [19]. Application of aqueous mixtures, replacing hazardous organic solvents, is one of the main strategies. Alternatively, natural deep eutectic solvents (NADES) have also been applied [12,20,21,22,23,24,25,26] for this purpose. In general, many multicomponent liquid mixtures, such as NADES [27,28,29,30,31,32], ionic liquids [30,33,34,35], and organic solvent mixtures [36,37,38] are considered as promising green solvents. Another reason for binary solvents research is the optimization of reactants concentrations and crystallization efficiency [39,40,41,42,43,44].

In the recent decade, the solubility of various sulfonamides in neat and binary solvents has been widely studied, both experimentally and theoretically [15,45,46,47,48,49,50,51,52,53,54,55,56,57,58,59,60,61,62,63,64,65,66,67,68,69,70]. However, in the case of sulfamethizole, only a few published solubility series are available. Data reporting multicomponent solvents (1,4-dioxane + water [71], methanol + water [56], propylene glycol + water [64,72]) are especially limited. Hence, this study fills this gap and extends the pool of available experimental solubility of sulfamethizole in neat and aqueous binary mixtures. The second goal of this study is to find green solvent alternatives by an extensive screening of a variety of solvent mixtures. Since it is impractical to measure the whole variety of solvent combinations, the machine learning protocol is used for the development of a solubility predictive model. Hence, the second aim of this study is the development of an accurate ensemble of neural networks model (ENNM), adequate both for backcomputations and screening of SMT solubility.

## 2. Materials and Methods

### 2.1. Materials

All chemicals used in this study were of analytical grade and were used without purification. Sulfamethizole (SMT, CAS: 144-82-1) and 1,4-dioxane (CAS: 123-91-1) were purchased from Sigma-Aldrich (Poznań, Poland). Acetonitrile (CAS: 75-05-08), N,N-dimethylformamide (DMF, CAS: 68-12-2), dimethyl sulfoxide (DMSO, CAS: 67-68-5), and methanol (CAS: 67-56-1) were obtained from Avantor (Gliwice, Poland). The nitrogen (grade 5.0) used in differential scanning calorimetry DSC measurements was obtained from Linde (Warsaw, Poland). All details were summarized in Table 1.

### 2.2. Sulfamethizole Solubility Determination

The solubility measurements were performed based on the shake-flask procedure reported in our previous papers [11,12,13,15]. First, the mixtures containing SMT solution and undissolved excess of solid were prepared in glass test tubes. For this purpose, 2000 µL of the solvent and appropriate amount of SMT were added to each tube. Then, the mixtures containing SMT solution and undissolved solid were placed in an Orbital Shaker Incubator ES-20/60 from Biosan (Riga, Latvia). The agitation speed was set to 60 rpm. After 24 h, the samples were filtered using preheated syringes and syringe filters (0.22 μm PTFE). Then, 100 µL of the filtrate was diluted in 2000 µL of methanol, while 500 µL was used for the pycnometric measurements carried out to determine the density of the solutions, which was necessary to determine the molar fraction solubility values. In all cases, the filtrate was collected using an automatic pipette with a preheated tip. The molar concentration of SMT in the samples was determined spectrophotometrically (λ_max_ = 284 nm) applying A360 UV-VIS device (AOE Instruments, Shanghai, China). In all cases, the samples were diluted with methanol, so that the absorbance was measurable and did not exceed the calibration curve range.

### 2.3. FTIR and DSC Characteristics of Solid Residues Obtained after Flask-Shake Procedure

After determining the solubility, the sediments remaining in the test tubes (in the case of pure solvents) were dried on air and subjected to Fourier transform infrared spectroscopy (FTIR) and differential scanning calorimetry (DSC) measurements. The FTIR spectra were recorded using the diamond attenuated total reflection (ATR) technique. For this purpose, a PerkinElmer (Waltham, MA, USA) spectrophotometer was used. DSC thermograms were determined using a DSC 6000 Perkin Elmer (Waltham, MA, USA) calorimeter. Nitrogen flow was set to 20 mL/min, and the heating rate was 5 K/min. The DSC device was calibrated using indium and zinc reference standards supplied by Perkin Elmer (Waltham, MA, USA). All measurements were performed in standard aluminum pans.

### 2.4. COSMO-RS Solubility Computations

The COSMO-RS (conductor-like screening model for real solvents) [73,74,75] is an approach used for studying neat or multicomponent bulk systems by taking advantage of both quantum chemistry and statistical thermodynamics. The part utilizing quantum chemical computations belongs to continuum solvation models in which physicochemical properties of the solute molecule are estimated using a molecule embedded in a perfect virtual conductor. The interface of molecular contact with environment is approximated by a discrete collection of segments of a given area, and the screening charge density was used for computation of interaction energies between closely packed molecules. In the second stage, such microscopic state properties are related to macroscopic thermodynamic properties by statistical thermodynamics [76]. The entire collection of surface pieces characterizing a liquid system is used for determination of the distribution function termed *σ*-profile, *P_s_*(*σ*′). In the case of mixtures, the *σ*-profile is summarized with concentration-dependent weighting. Such distribution functions are used for derivation of the *σ*-chemical potential, *μ_S_*(*σ*), by iteratively solving the exact equation:

(1) $$\mu_{s}\left(\sigma \right)=-\frac{RT}{a_{aff}}ln\left[\int^{\;}P_{s}\left(\sigma^{\prime }\right)exp\left\{\frac{a_{aff}}{RT}\left[\mu_{s}\left(\sigma^{\prime }\right)-e\left(\sigma ,\sigma^{\prime }\right)\right]\right\}d\sigma^{\prime }\right]$$

where *μ_S_*(*σ*) represents the chemical potential of an average molecular contact area of size *a_eff_* in the ensemble *S* at temperature *T*, $e\left(\sigma ,\sigma^{\prime }\right)$ is the sum of the three (misfit, hydrogen bonding, and dispersion) contributions to the intermolecular interaction. The resulting integral function defined in Equation (1) enables complete description of the thermodynamics of the system including the residual part of the chemical potential. It is essential to note that *μ_S_*(*σ*) contains the crucial representation of molecular interactions [76]. The *μ_S_*(*σ*) distribution is typically provided in a discrete representation as a set of 61 points in the range of charge density between *σ* = ±0.03 e/Å^2^. However, heuristic analysis [76] suggests that three fundamental regions are to be distinguished. Indeed, regions *σ*∈<−0.03,−0.01> characterize affinity for HB donors (HBD), the range *σ*∈<−0.01,0.01> characterizes nonpolar interactions and is regarded as a measure of hydrophobicity (HYD), and the high positive polarity interval *σ*∈<0.01,0.03> quantifies affinity for HB acceptors (HBA). Since the whole 61-point *μ_S_*(*σ*) distribution possesses redundant information, data reduction is to be applied prior to the practical application as molecular descriptors used for machine learning purposes. Here, a simple approach was adopted by averaging *μ*(*σ*) values within Δ*σ* = 0.02 regions. Hence, the resulting six descriptors can be summarized as follows:

(2) $$spot1=\bar{\mu }\left(\sigma \in \langle -0.03,-0.02\rangle \right);\;spot2=\bar{\mu }\left(\sigma \in \langle -0.02,-0.01\rangle \right);\;\;spot3=\bar{\mu }\left(\sigma \in \langle -0.01,\;0.00\rangle \right);\;spot4=\bar{\mu }\left(\sigma \in \langle 0.00,+0.01\rangle \right)\;spot5=\bar{\mu }\left(\sigma \in \langle +0.01,+0.02\rangle \right);\;spot6=\bar{\mu }\left(\sigma \in \langle +0.02,+0.03\rangle \right)$$

It is also worthwhile to further group the above descriptors into three categories:

(3) HBA=μ(σ∈〈−0.03,−0.01〉)=spot1+spot2HYD=μ(σ∈〈−0.01,+0.01〉)=spot3+spot4HBD=μ(σ∈〈+0.01,+0.03〉)=spot5+spot6

It is worth mentioning that, for the practical calculations of these properties, a proper representation of the molecular structure is indispensable, both in the gas and condensed phases. For this purpose, COSMOconf is used for generation of the most energetically favorable conformations. This program performs quantum chemistry calculations using TURBOMOLE rev. V7.5.1 (Karlsruhe, Germany) interfaced with BIOVIA TmoleX 2021 (version 21.0.1, BIOVIA, San Diego, CA, USA). The level of theory used at this stage corresponded to RI-DFT BP86 (B88-VWN-P86) with def-TZVP basis set for geometry optimization and def2-TZVPD basis set for single point calculations with inclusion of the fine grid tetrahedron cavity and inclusion of parameter sets with hydrogen bond interaction and van der Waals dispersion term based on the “D3” method of Grimme et al. [77]. This method of computations is further referred to as the BP level. All of the solubility calculations were performed using COSMOtherm (version 20.0.0, revision 5273M, BIOVIA, San Diego, CA, USA) [78] with BP_TZVPD_FINE_20.ctd parametrization.

Pairs formation was assessed by computing the affinity of SMT for the solvent molecule using a standard thermodynamic cycle. The same level of computations was used as for other types of computations but augmented with correction for zero point vibrational energy ZPE. Hence, the values of Gibbs free energies of reaction A + B = AB (A = SMT, B = solvent molecule) were computed using a concentration-independent protocol offered by COSMOtherm. Affinities of SMF dimers formation were computed in a similar manner.

### 2.5. Affinity Indices

Molecular descriptors coming from simplified potentials (after data reduction) were used for quantification of solute–solvent affinities. Three major contributions can be distinguished coming from specific and nonspecific interactions. The former can be attributed to hydrogen bonding of the solute molecule, which can act either as a donor or acceptor with solvent molecules, offering its acceptor or donor sites, respectively. For nonspecific interactions, the low polar regions of molecular centers should be considered. Hence, mutual affinities can be defined by introducing the following indices:

- DA index as the measure of mutual affinity of HB donor of solute (HBD_solute_) and HB acceptor of the binary solvent (HBA_solvent_), DA = HBD_solute_ − HBA_solvent_.
- AD index as the measure of mutual affinity of HB acceptor of solute (HBD_solute_) and HB donor of the binary solvent (HBA_solvent_), AD = HBA_solute_ − HBD_solvent_
- HH stands for hydrophobicity measure, HH = HYD_solute_ − HYD_solvent_
- Affinity complementarity index is simply the sum of the three above, AC = AD + DA + HH.

### 2.6. Machine Learning Protocol

The machine learning was conducted in two stages. Initially, Statistica software, TIBCO Software Inc., Palo Alto, CA, USA (version 13) was used for Statistica Automated Neural Networks (SANNs) growth. In this study, default SANN settings were assumed. This includes one layer architecture, multilayer perceptron (MLP), 70:15:15 data set splitting into training, validation, and test sets, and the sum of squares (SOS) error function. For the input layer, six COSMO-RS descriptors were used. The output layer was the logarithm of molar fraction solubility. The second stage involved successful accumulation of networks fulfilling the following formal criterions of SANN acceptance:

1. accuracy: RMSD < 0.035 (root mean square deviation);
2. precision: number of outliers out of domain ≤3 (of 175), ~less than 1.7%;
3. reliability: predicted solubility within the formal range of log(*x*) between 0 and 1 for at least 99% of predicted or backcomputed values.

In order to evaluate the applicability domain, the well-known protocol based on h* statistics was used [79,80,81].

## 3. Results and Discussion

The organization of the paper reflects the steps undertaken for realization of the desired goals. First, the data set of sulfamethizole solubility was collected by new measurements in five aqueous binary mixtures with organic solvents. Then, an ensemble of artificial neural networks, ENN, was developed, taking advantage of molecular descriptors characterizing *σ*-sigma potentials. Finally, extensive screening was performed for finding new promising binary solvents as potential solubility enhancers of SMT. Particular attention was paid to the green nature of solvents.

### 3.1. Sulfamethizole Solubility

**Figure 1:**
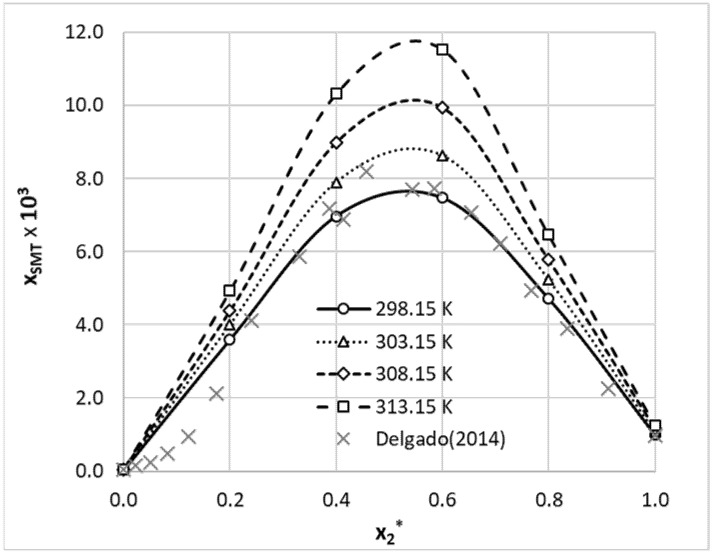
Molar fraction solubility of sulfamethizole in aqueous 1,4-dioxane binary mixtures. On the ordinate, *x*_2_* represents the mole fraction of organic solvent in solute-free binary solution. The available literature values published by Delgado in 2014 [71] for 298.15 K were marked with gray crosses.

**Figure 2:**
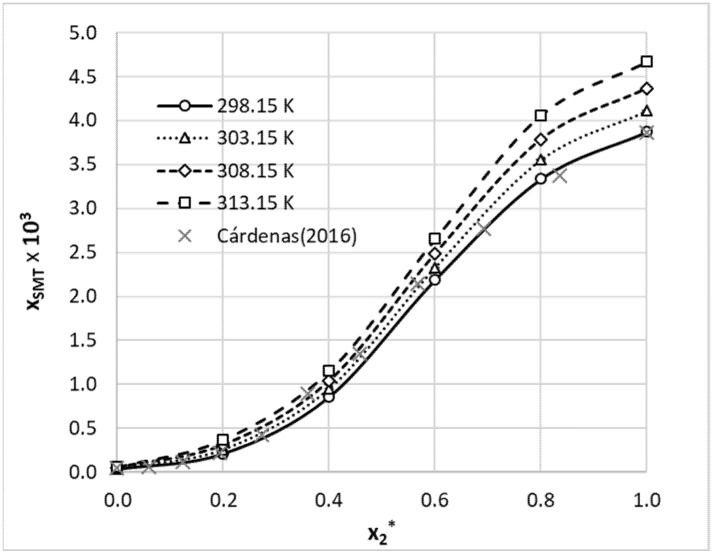
Molar fraction solubility of sulfamethizole in aqueous methanolic binary mixtures. On the ordinate, *x*_2_^*^ represents the mole fraction of organic solvent in solute-free binary solution. The available literature values published by Cárdenas in 2016 [56] for 298.15 K were marked with gray crosses.

The starting point of this study was the augmentation of a limited pool of available solubility data of SMT with new measurements. Aqueous binary systems were selected due to the most probable practical implications. The obtained results were collected in Table 2. Additionally, the solubility data for aqueous solutions of SMT in 1,4-dioxane and methanol were presented graphically (Figure 1 and Figure 2) for comparison of our results with already published ones [56,71]. As can be inferred from Figure 1 and Figure 2, solubility trends of this paper are quite consistent with previously reported data. The solubility profiles of the rest of the measured systems were collected in the Supporting Materials (see Figures S1–S3). From provided data, it is clearly visible that SMT is poorly soluble in water and, at room temperature, solubility is as low as *x*_SMT_ = 3.4 × 10^−5^. Hence, it is not surprising that any of the utilized organic solvents can act as an efficient cosolvent with the highest solubility enhancement observed in the case of DMF and DMSO. For these organic solvents, the solubility advantage, defined as the logarithm of molar fraction solubility,

(4) $$SA=log\left(\frac{\log(x_{\mathrm{SMT}}\left(\mathrm{organics},T=298.15\;K\right)}{x_{\mathrm{SMT}}\left(\mathrm{water},\;T=298.15\;K\right))}\right)$$

is as high as 3.8 and 3.7, respectively. The values of *SA* for 1,4-dioxane and acetonitrile are much lower and are equal to 1.5 and 1.9, respectively. Utilization of methanol as a solvent results in enhancement of solubility by about two orders of magnitude compared to the solubility provided by water (*SA* = 2.1). The solubility advantage offered by propylene glycol is also comparable (*SA* = 2.3), which can also be inferred from published data [64,72]. Hence, water can be regarded as an efficient antisolvent for any of the studied solvents, which might be used for recrystallization purposes. It is also interesting to note that 1,4-dioxane exhibits a synergistic effect, with the highest solubility corresponding to *x*_2_^*^ = 0.6 (*x*_2_* represents the mole fraction of organic solvent in solute free binary solution). In such a composition, solubility of SMT is 220 times higher than in pure water (*SA* = 2.3) and exceeds the solubility in neat 1,4-dioxane by about seven times. In the case of an acetonitrile–water system, a similar cosolvation behavior can be observed. The highest solubility advantage, *SA* = 2.55, can be observed for *x*_2_* = 0.6. Hence, both the 1,4-dioxane–water system and the acetonitrile–water system can offer additional benefits worth consideration in practical applications. In the case of methanol–water solvents, moderate deviations from the linear trend can be observed for both low and high organic solvent contributions in the binary mixture.

Although it is interesting to notice a high solubility of SMT in DMF, this particular solvent is regarded as hazardous and reprotoxic with restriction consideration imposed by the European Chemicals Agency’s (ECHA) Registration, Evaluation, Authorization and Restriction of Chemicals (REACH) [82]. Hence, utilization of this solvent in the pharmaceutical industry is seriously limited. Fortunately, the second best solvent found for sulfamethizole, DMSO, does not undergo such serious restrictions and offers comparable solubility of SMT. In this case, the solubility enhancement is about 5300 times higher compared to water at room temperature. According to several reports, DMSO is considered as a green solvent [83,84,85]. Noteworthily, DMSO has been widely applied in the pharmaceutical industry [86]. Furthermore, this compound is listed in the DrugBank database [87,88] and exhibits analgesic, antioxidant, and anti-inflammatory activities. The beneficial properties of DMSO, as a pharmaceutical excipient, are associated with the skin permeability enhancement capabilities. Noteworthily, both sulfonamides and DMSO have been used for the treatment of dermatological diseases [89,90,91,92]. This coincidence appears to be of interest in the context of considering the sulfamethizole–DMSO system as a pharmaceutical formulation candidate.

Since the aim of this work is to develop a solubility model of SMT based only on the COSMO-RS solution characteristics, it is important to determine whether the solid state that is in equilibrium with liquid has not undergone any polymorphic or pseudopolymorphic transformations. For this purpose, the FTIR and DSC measurements were carried out for the solid residues obtained after the shake-flask solubility determination procedure was performed for neat solvents. Fortunately, in all cases, both IR spectra and DSC thermograms for precipitates are similar to those recorded for pure SMT (see Figure S4 in Supplementary Materials).

### 3.2. Predictive Solubility Model

**Figure 3:**
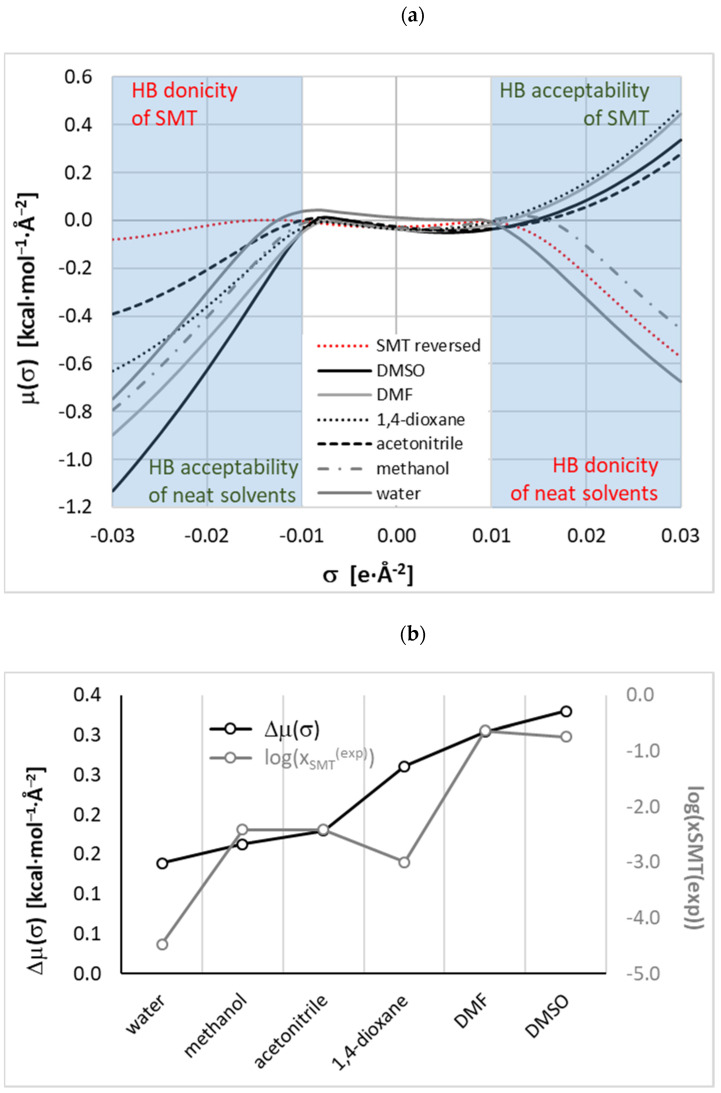
(**a**) Distributions of *σ*-potentials, *μ*(*σ*), as a function of charge density, *σ*, for six neat solvents and SMT at room temperature. Trend of the solute was presented in the reversed form. (**b**) Qualitative correspondence between solubility (gray lines and right axis) and TA index for studied systems.

From the provided experimental data, it was concluded that after excluding DMF due to its nongreen character, DMSO becomes the first choice solvent for SMT. On the other hand, in the literature, there were many examples [19,93,94] of replacements of hazardous solvents with ones of lesser toxicity and more environmental friendliness. It is interesting to see if there is any replacer for DMF also exhibiting such high solubility. For this purpose, nonlinear modeling was used with the methodology similar to already applied for solubility screening of theophylline [12]. This method relies on the machine learning protocol applied for development of an ensemble of neural networks (ENN). In this approach, a series of artificial neural networks fulfilling the inclusion criteria are collected and used for final solubility predictions. The main difference between the former work [12] and this paper is in the type of information used for machine learning. Here, a much simpler and more intuitive set of molecular descriptors was used. They come from sigma potentials, *μ*(*σ*), computed according to COSMO-RS theory [95] with an aid of COSMOtherm software [78]. In Figure 3, the distributions of *μ*(*σ*) as a function of charge density were plotted for solvents used in this study. The analysis also includes sulfamethizole in aqueous binary solvents containing propylene glycol for which the solubility values have been documented by Delgado et al. [64,72]. Additionally, a reversed trend of sulfamethizole was also added for comparison. Such a method of presentation allows for direct qualitative analysis of putative intermolecular interactions due to hydrogen bonding. This is supposed to be the dominant factor in the case of systems with proton-accepting and proton-donating centers. As is commonly recognized [74,95], the affinity for hydrogen bonding donors, HB accepting ability, corresponds to negative charge density regions, and vice versa—the affinity for hydrogen bonding acceptors, HB donating ability, corresponds to positive charge densities. Hence, a lower value of *μ*(*σ*) in Figure 3 corresponds to a stronger affinity of a given type. The reversing trend used for the solute enables inspection of the direct match with solvent molecules via complementary centers. In other words, in Figure 3a, a higher distance between SMT plots and the ones characterizing a given solvent corresponds to a higher overall HB tendency of solute–solvent interactions, which might indicate higher solubility. Indeed, in Figure 3b, two plots showing interesting correspondence were presented. The gray line, representing solubility, is associated with the right ordinate. The second line drawn in black color denotes the area between *μ*(*σ*) of solvent with respect of solute and is associated with the left ordinate. Both lines represent quite similar trends allowing for qualitative ranking of solvents. Two the most efficient solvents might be properly selected for experimental tests, even though such inference is only qualitatively correct. Nevertheless, there is a quite rational expectation that information provided by *μ*(*σ*) functions might be used as valuable molecular descriptors for machine learning protocol.

**Figure 4:**
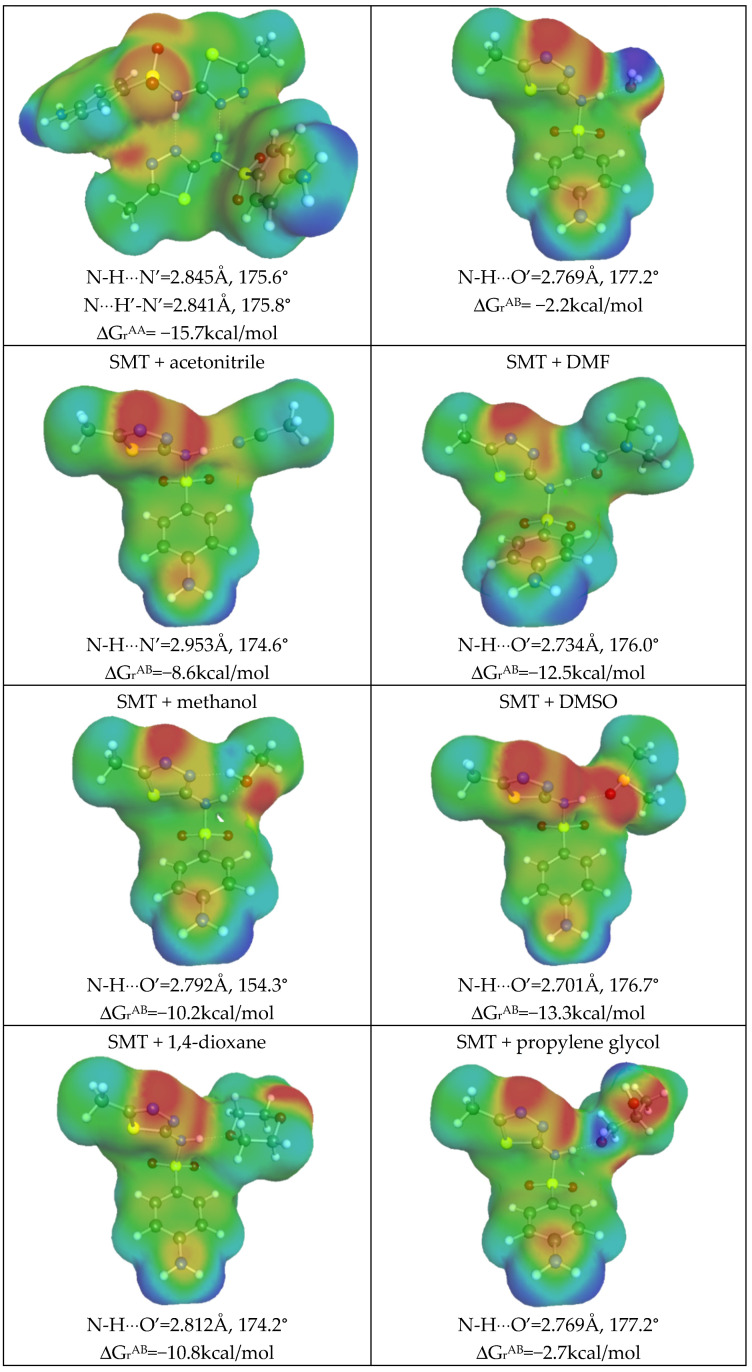
Schematic representation of structure and charge densities of the most stable pairs involving sulfamethizole in studied systems. ΔG_r_ values represent concentration-independent pairs affinity commutated at the BP level.

From Figure 3a, it can be inferred that HB donating potential of SMT is rather modest compared to water or methanol, for which it is expected to be the highest among studied systems. This property of SMT is granted from the hydrogen atom attached to the nitrogen center located in the amide linkage. The propensity of SMT for hydrogen bonding is strong enough for dimerization, as is documented in Figure 4. SMT is rich in electronegative centers, but it is a rather weak HB acceptor due to positive values of potential in the region of *μ*(*σ*) positive values of charge density distribution. To the contrary, it acts as a proton donor with all considered solvents molecules. The schematic representations of the most energetically favorable structures are characterized in Figure 4. It is clearly visible that the hydrogen bonding pattern is the same for all pairs. Hydrogen bonds are short with almost perfectly open angles between hydrogen H-N covalent bonds of SMT. Additionally, the strong nature of formed hydrogen bonds is confirmed by the value of Gibbs free energy of pairs formation. As was mentioned in the methodology part, the affinity values are computed as concentration-independent activity equilibrium constants of SMT-X molecular complex formation. In the case of a dimer, X = SMT; otherwise, the solvent molecule is represented by the X symbol. All heteromolecular pairs are also probable in aqueous solutions, which is indicated by ΔG_r_ values provided in Figure 4, which also indicate much stronger affinity of SMT to organic solvents rather than water. This might be the reason of low solubility of SMT in neat water. It is also not surprising that the strongest complexes of SMT are formed with DMSO and DMF. Again, a qualitative relationship is obtained between SMT affinities for solvent molecules and observed solubility. Unfortunately, there are no linear relationships between these data, and that is why ENN was developed for precise solubility backcomputations and predictions.

**Figure 5:**
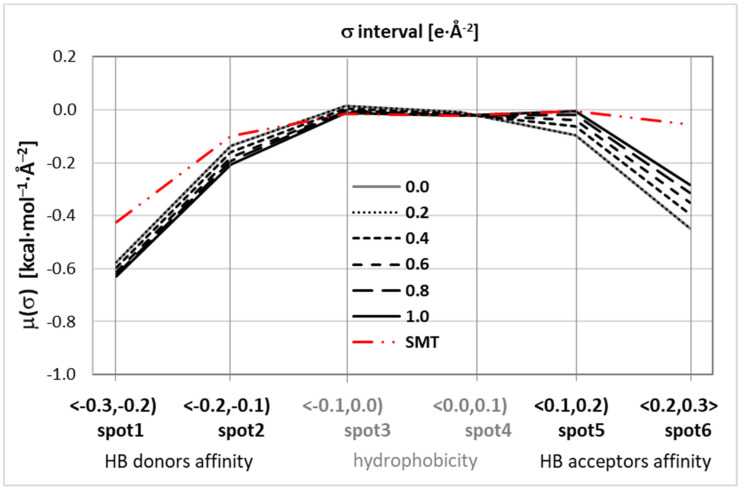
Distributions of the values of descriptors characterizing SMT in aqueous methanol binary mixtures at room temperature. Series correspond to systems differing in mole fraction of organic solvent.

Machine learning protocol utilized the distributions of *μ*(*σ*), which, after data reduction, resulted only in six molecular descriptors per system. The representative distributions of these six measures were presented in Figure 5 for methanol–water solutions at room temperature in six compositions. The rest of the studied systems were characterized in the Supporting Materials (see Figures S5–S9). As can be inferred from Figure 5, the *μ*(*σ*) profiles of protic solvents (methanol, propylene glycol) are significantly different from the ones corresponding to aprotic media (DMF, DMSO, 1,4-dioxane, acetonitrile). This effect is particularly pronounced in the case of neat solvents (1.0), as evidenced by an upward trend for large *σ* intervals (HB acceptors affinity area) for aprotic solvents and an opposite downward trend for protic ones.

**Figure 6:**
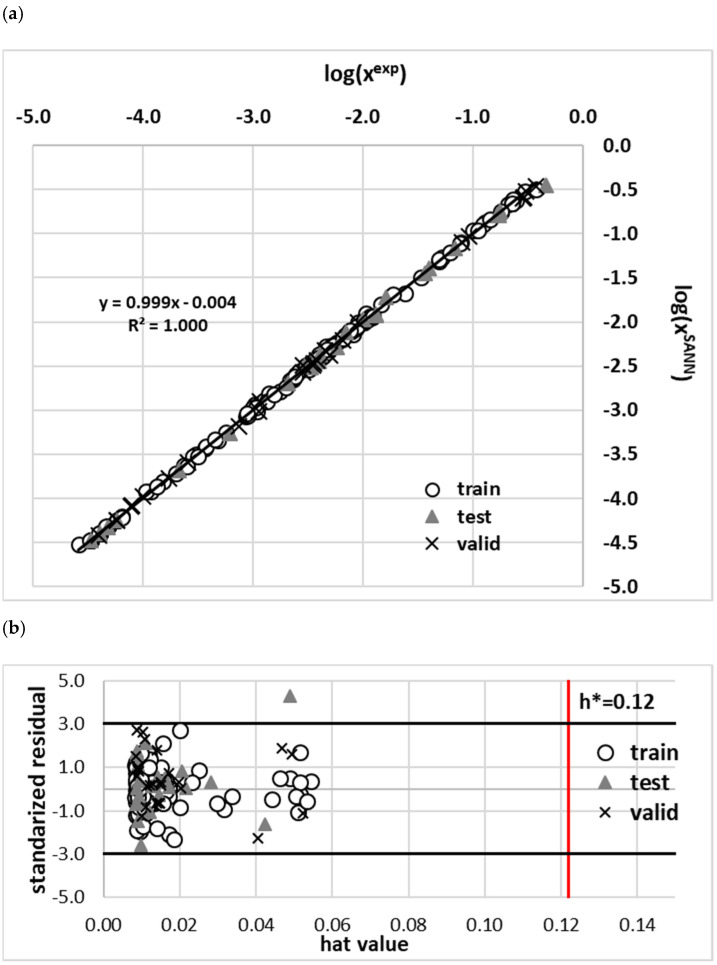
Representative example of sulfamethizole solubility prediction using one of the neural networks included in the developed ensemble of SANNs (top panel **a**) along with the applicability domain (bottom panel **b**) for 175 data points.

**Figure 7:**
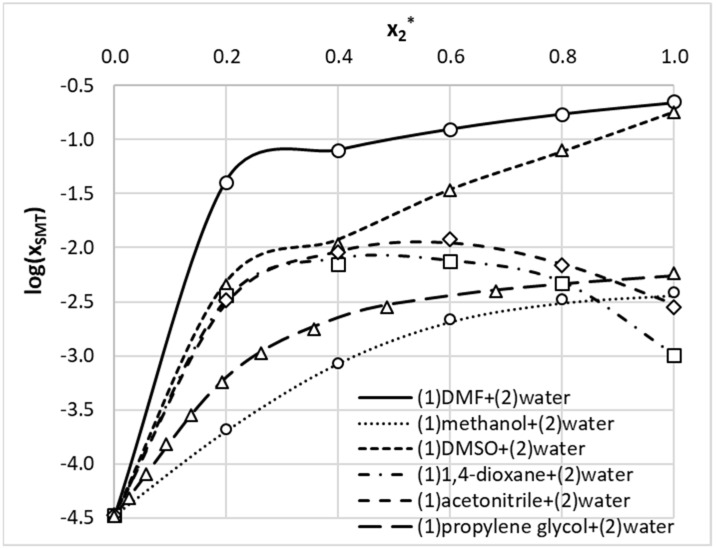
Accuracy of sulfamethizole solubility prediction using the developed ensemble of SANNs applied for five studied binary systems at room temperature. Open symbols represent measured data; the lines stand for predicted trends. *x*_2_* represents the mole fraction of an organic solvent in solute-free binary solution.

**Figure 8:**
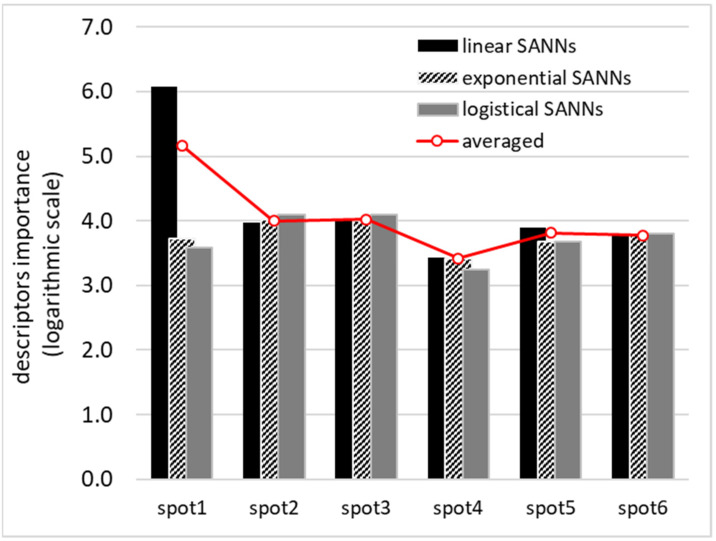
Results of sensitivity analysis providing information about importance of the descriptors distributions.

The ENN was constructed by successful collection of SANNs fulfilling the acceptance criterions. Since accuracy expressed in terms of RMSD was not the only inclusion criterion, it is expected that obtained ENN is sufficiently coherent for predictive purposes. The quality of obtained ENN was documented in Figure 6. The applicability domain was characterized in the form of a relationship between standard residuals and hat values. There is almost a perfect match between backcomputed solubility values for the set of 175 data points and experimental ones. For further documentation of the accuracy of the developed model, SMT solubilities in studied systems were plotted in Figure 7. The developed ENN is characterized in greater detail in the Supporting Materials (see Table S1). It is worth mentioning that the obtained ENN is quite heterogeneous, which can be inferred from the fact that diverse neural networks were included in the final ensemble differing in mathematical formulations. Indeed, the tanh function was used as an activation in 91% of included SANNs and logistic functions was implemented in remaining 9%. About 62% of networks included in the ENN utilized a linear output function, 33% were constructed based on an exponential function, and only 5% were constructed based on a logistic function. It is also interesting to note that all molecular descriptors made significant contributions to the final ENNM. This can be inferred from the sensitivity analysis provided in Figure 8. It is directly visible on the provided plots that all three regions of *μ*(*σ*), characterizing HB accepting and HB donating abilities and hydrophobicity, are utilized in SANN development. It seems that the contribution coming from HB accepting ability is slightly more pronounced, which was already addressed by inspection of the potential occurrence of intermolecular complexes.

### 3.3. Sulfamethizole Solubility Screening

The accuracy of developed ENN encourages prediction of SMT solubility for systems not studied experimentally. This was performed via computations of molecular descriptors values for a variety of binary mixtures comprising combinations of 180 solvents used in practice for solubility determination. The list of solvents comes from the in-house database of solubility data published in the literature. From the perspective of the aim of this paper, binary mixtures are the most interesting. However, it is not practical to test all possible combinations of neat solvents given the restriction not from the computational perspective but from that of the potential miscibility limitations. In order to avoid studying artificial combinations, which, in practice, might result in binary biphasic systems, only pairs of miscible liquids were considered. This was ensured by an additional literature search. Hence, for the screening purposes, 275 binary systems were studied in six compositions at room temperature. Additionally, the pool of considered solvents was extended by including solvents suggested by the PARISIII application [96,97,98,99,100] as potential greener alternatives for two solvents with the highest solubility of SMT. This software was developed by the U.S. Environmental Protection Agency (EPA) [101] and was designed mainly for screening for more environmental friendly solvents, which can potentially replace problematic ones. Hence, neat and aqueous binary mixtures of DMF or DMSO were included in the search for greener alternatives. All aqueous binary composition considered for experimental solubility measures were used as the initial mixtures for PARISIII inputs. All solvents classified in the program as green ones were used in the screening phase. This is a somewhat laborious procedure due to the lack of automatic mechanisms offered by the current version of the software. Hence, this procedure was repeated for every initial mixture, and as a result, one hundred suggested binary solvent mixtures in compositions proposed by the program were collected. As a result, this phase seriously extended the pool of considered solvents used for SMT solubility screening.

**Figure 9:**
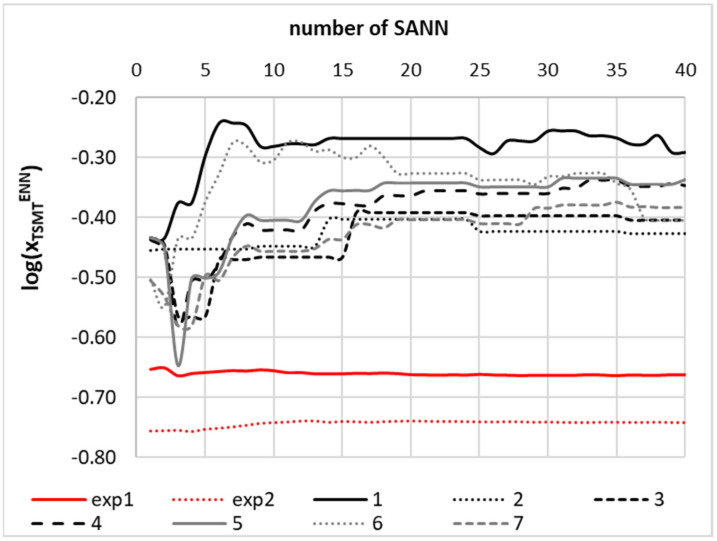
Results of solubility screening with an aid of developed ENN. Individual SANNs are sorted with rising RMSD, and values are averaged systematically, including increasing number of SANNs. The mean value predicted by ENN corresponds to number 40. The following systems are presented: exp1: DMF, exp2: DMSO, 1: 4-formylmorpholine, 2: formamide, 3: N-methylformamide, 4: DMF + formamide (*x*_2_* = 0.8), 5: DMF + N-methylformamide (*x*_2_* = 0.4), 6: methanol + formamide (*x*_2_* = 0.4), 7: methanol + N-methylformamide (*x*_2_* = 0.2).

For each solvent included in the final list, the values of six molecular descriptors were determined analogically to the training set and were used as inputs for the development of the ENNM. Estimated SMT solubilities were confronted with solubility in DMF to find solvents with comparable or better effectiveness. It is interesting to summarize that during this phase, several systems were identified as potential solubility enhancers of SMT. The results of the solubility computations for selected binary systems are provided in Figure 9. The presented values are computed by successful averaging with inclusion of an increasing number of SANNs, which were sorted according to increasing values of RMSD. Hence, the presented trend starts with prediction of the most precise SANN and ends on the values averaged over all networks constituting the entire ENNM. It is visible that stable predictions are provided for the majority of systems including backcomputed values for SMT solubility in neat DMF and DMSO. In these cases, few SANNs are indispensable for convergence of predicted solubility values. In other cases, a more extended set of SANNs is necessary for stabilizing the mean values. At least 20 networks are necessary in the majority of cases. It is worth mentioning that extension of the number of SANNs constituting ENN is straightforward and not time-consuming. Hence, it does not stand as a limiting factor due to automation of the whole procedure of ENNM production. As is documented in Figure 9, three neat solvents (4-formylmorpholine, formamide, and N-methylformamide) were identified as more efficient SMT solubilizers compared to DMF. The model found 4-formylmorpholine as the solvent with the highest solubility potential. It is worth noting that 4-formylmorpholine has been already used as a green solvent for solid phase peptide synthesis [102,103] and in patented agricultural formulations [104]. The only problem with this solvent is its high melting temperature, which is close to ambient conditions (MP = 294 K). The other two, N-methylformamide and formamide, are not classified as green solvents [105,106]. For more detailed characteristics of this aspect, all of the most interesting solvents were evaluated using PARISIII. The screening results were presented in the Supplementary Materials in Table S2. According to the overall environmental safety expressed by the environmental index (EI) provided in parenthesis, the considered solvents can be ranked in the following order: water (0.020) < 4-formylmorpholine (0.509) < N-methylformamide (0.959) < methanol-N-methylformamide (*x*_2_* = 0.2) mixture (1.071) < acetonitrile–water (*x*_2_* = 0.6) mixture (1.461) < DMF-N-methylformamide (*x*_2_* = 0.4) mixture (1.500) < acetonitrile (1.881) < methanol (1.893) < DMF (2.156) < methanol-formamide (*x*_2_* = 0.4) mixture (2.164) < DMF-formamide (*x*_2_* = 0.8) mixture (2.174) < formamide (2.295) < propylene glycol (4.499) < 1,4-dioxane–water (*x*_2_* = 0.6) mixture (4.633) < 1,4-dioxane (5.267) < DMSO (11.660). The unexpected scoring of DMSO, which is generally considered as a safe solvent, is worth commentary. According to the algorithm applied in the PARISIII program, DMSO was ranked as the least green solvent among all of the solvents mentioned above. This counterintuitive conclusion originates from the fact that the default settings assume equal contribution of all environmental impact scores to the overall environmental index. The only serious environmental aspect of DMSO is related to the extremely high value of the photochemical oxidation potential index (PCOP). However, from the perspective of pharmaceutical practice, this index seems to be of minor importance. If PCOP is excluded from the analysis for the re-evaluation of the environmental index values, the following series is obtained: water (0.020) < propylene glycol (0.189) < DMSO (0.260) < 4-formylmorpholine (0.509) < methanol (0.763) < 1,4-dioxane–water (*x*_2_* = 0.6) mixture (0.853) < methanol-N-methylformamide (*x*_2_* = 0.2) mixture (0.936) < N-methylformamide (0.959) < 1,4-dioxane (0.967) < acetonitrile–water (*x*_2_*=0.6) mixture (1.461) < DMF-N-methylformamide (*x*_2_* = 0.4) mixture (1.500) < methanol-formamide (*x*_2_* = 0.4) mixture (1.801) < acetonitrile (1.881) < DMF (2.156) < DMF-formamide (*x*_2_* = 0.8) mixture (2.174) < formamide (2.295). It is worth noting that, regardless of the total environmental index evaluation, 4-formylmorpholine is the top ranged solvent and can be regarded as a green alternative for DMF.

**Figure 10:**
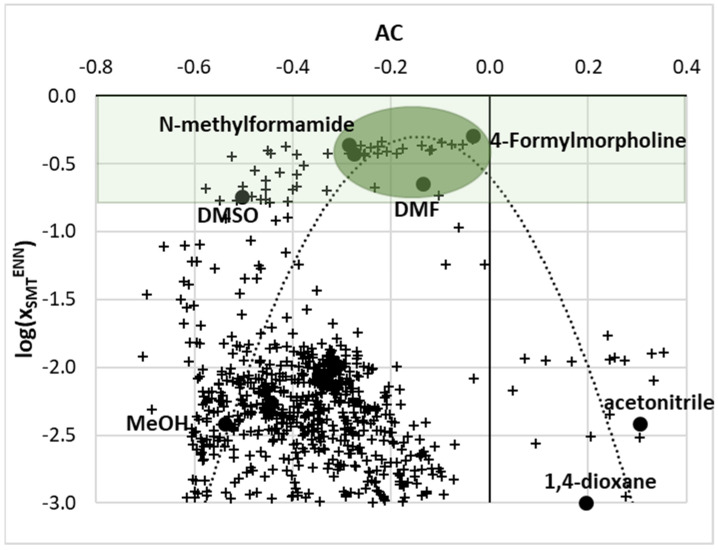
Correlation of the computed values of SMT solubility and values of the affinity complementarity index.

To complete the screening, an additional analysis was performed. The values of SMT solubility predicted using ENNM were plotted as a function of the affinity complementarity index. As was mentioned in the methodology section, AC is the sum of the relative acceptor, donor, and nonpolar indices describing the overall similarity of SMT affinity profiles with respect to a given solvent molecule. The cloud of points was generated using ENNM for hundreds of solvent mixtures at room temperature, as shown in Figure 10, where the distribution of AC was presented as the function of computed solubility. It is interesting to note that one can identify a high solubility zone, marked as a green region, within which all previously discussed SMT solubilizers are located, including DMSO and DMF. However, restricting interests only to the part of the green zone, which is characterized by close to zero values of the AC region, one can find the systems exhibiting the highest solute–solvent complementarity. This was marked with a green oval. It is quite understandable that small values of AC suggest high complementarity of *μ*(*σ*) profiles, which is a good indicator of potential solubilizing abilities. There are many potential binary systems with solubility advantages similar to that of DMF and the majority of them comprise DMF, DMSO, 4-formylmorpholine, formamide, and N-methylformamide in binary mixtures with themselves or other solvents such as water or light alcohols. It is also worth adding that all systems for which the values were plotted in Figure 10 belong to the applicability domain. Here, the critical hat value computed for training set is equal to h* = 0.122. All systems used in the screening procedure for which the computed hat value exceeded this threshold were excluded from the analysis. In this group, binary mixtures involving nonpolar solvents such as cyclohexane, toluene, benzene, and other hydrocarbons were found in a variety of binary formulations. This is rather expected due to character of the data set used at the training stage. Halogenated solvents were typically rejected from the analysis—for example, chloroform, carbon tetrachloride, and chlorocyclohexane mixed with other solvents. Additionally, promising green hydrotropes such as dihydrolevoglucosenone, gamma-valerolactone, sulfolane, glyme, diglyme, and transcutol were also identified as unsuitable for the detailed analysis due to high hat values. Some esters were located outside of the applicability domain—for example, ethyl acetate and propyl acetate. However, some surprising exclusions were found—for example, DMSO mixture with ethanol (*h* = 0.19, *x*_2_* = 0.8) or 2-propanol (*h* = 0.18, *x*_2_* = 0.6), as well as some light alcohols mixtures such as methanol + ethanol and methanol + propanol. This is probably due to the limited diversity of the training set of SMT solubility data. Identification of formally acceptable solubility enhancers compensated for these surprising exclusions.

**Figure 11:**
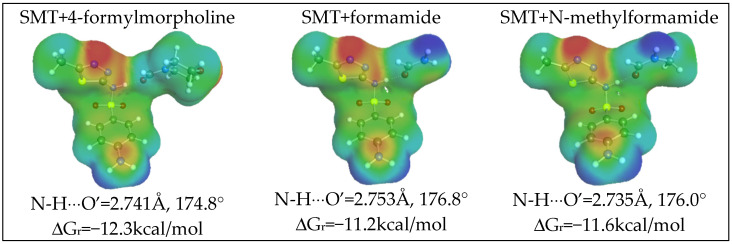
Schematic representation of structure and charge densities of the most stable pairs involving sulfamethizole in the studied systems. The notations are the same as those in Figure 4.

Finally, it is also interesting to provide information about the affinities of SMT for the solvents found during the screening phase. Hence, in Figure 11, structural land energetic data are presented along with graphical representations of charge density distributions. A summarization of all computed affinities is also provided in Figure 11. It is clearly visible that the formyl group can act as an efficient hydrogen bonding acceptor due to the electronegativity of the oxygen atom. The higher solubilizers of SMT are characterized by the highest values of SMT affinity for formation of heteromolecular pairs with solvent molecules.

## 4. Conclusions

The search for efficient and green solvents is a general tenet of the sustainable chemistry concept. This is as important, as it is a not trivial and not straightforward task. The necessary compromise between often contradictory constraints prohibits easy replacement of hazardous solvents with greener ones. In this study, the general approach for this task is offered with quite spectacular success. Here, in the case of sulfamethizole, similarly to the already documented case of theophylline [12], the ensemble of neural networks concept was implemented for not only backcomputation of experimental data but also for efficient screening purposes. Carefully controlling hat values enables exclusion of systems not belonging to the applicability domain. The efficient utilization of the machine learning protocol requires an adequate pool of experimental data.

Since the knowledge of SMT solubility was too limited, the results of new measurements were provided for five aqueous binary systems. This analysis was enriched with green solvents screening procedure based on the several common environmental risks assessment. The application of water–organic mixtures seems to be a promising strategy in seeking greener solvents. For instance, two of the binary water–organic mixtures, 1,4-dioxane–water (*x*_2_* = 0.6) and acetonitrile–water (*x*_2_* = 0.6), were found to be more efficient and were ranked as more environmentally friendly than pure organic components.

In this study, the range of SMT solubility values was extended to include much more effective solvents. Following the performed experiments, the high solubilizing potentials of DMF and DMSO were documented. Since the former solvent cannot be used in pharmaceutical practice, the search was undertaken for greener replacement with high solubility enhancement. The application of ENN enabled finding real alternatives for DMF with even higher solubilizing power. Hence, finding 4-formylmorpholine is the main outcome of this study, showing the efficacy of the proposed approach.

## Figures and Tables

**Table 1:** The characteristics of chemicals used in the study.

IUPAC Name	CAS Code	Vendor	Initial Purity (Mass Fraction)	Purification Method
4-Amino-N-(5-methyl-1,3,4-thiadiazol-2-yl)benzenesulfonamide (SMT)	144-82-1	Sigma-Aldrich (Poznań, Poland)	≥0.99	none
1,4-Dioxane	123-91-1	Sigma-Aldrich (Poznań, Poland)	0.998	none
Acetonitrile	75-05-08	Avantor (Gliwice, Poland)	≥0.995	none
N,N-Dimethylformamide (DMF)	68-12-2	Avantor (Gliwice, Poland)	≥0.998	none
(Methylsulfinyl)methane (DMSO)	67-68-5	Avantor (Gliwice, Poland)	≥0.997	none
Methanol	67-56-1	Avantor (Gliwice, Poland)	≥0.998	none
Nitrogen	7727-37-9	Linde (Warsaw, Poland)	0.99999	none

**Table 2:** Values of experimentally determined sulfamethizole solubility in five studied aqueous organic solvents binary mixtures. The first column comprises mole fractions of organic solvent in solute free solutions. (*x*_2_* represents the mole fraction of organic solvent in solute-free binary solution).

*x*_2_*	298.15 K	303.15 K	313.15 K	313.15 K
1,4-Dioxane + water, *x*_SMT_ × 10^4^				
0.0	0.34 ± 0.01	0.41 ± 0.02	0.48 ± 0.01	0.58 ± 0.02
0.2	36.00 ± 1.06	40.20 ± 1.61	43.88 ± 2.51	49.37 ± 2.90
0.4	69.74 ± 2.14	79.01 ± 2.74	89.97 ± 2.50	103.25 ± 2.35
0.6	74.69 ± 3.14	86.23 ± 2.56	99.46 ± 3.01	115.37 ± 3.04
0.8	47.15 ± 1.50	52.31 ± 1.66	57.73 ± 2.33	64.57 ± 2.94
1.0	10.03 ± 0.32	10.79 ± 0.39	11.63 ± 0.64	12.49 ± 0.57
Methanol + water, *x*_SMT_ × 10^4^				
0.2	2.12 ± 0.13	2.57 ± 0.14	3.06 ± 0.12	3.69 ±0.23
0.4	8.58 ± 0.43	9.50 ± 0.38	10.36 ± 0.48	11.50 ± 0.58
0.6	21.88 ± 0.68	23.29 ± 0.68	24.86 ± 0.56	26.53 ± 0.57
0.8	33.36 ± 0.68	35.54 ± 0.82	37.89 ± 0.78	40.62 ± 1.12
1.0	38.72 ± 0.91	41.10 ± 1.35	43.68 ± 1.30	46.70 ± 1.20
DMF + water, *x*_SMT_ × 10^2^				
0.2	3.99 ± 0.21	4.99 ± 0.26	6.19 ± 0.21	7.68± 0.17
0.4	8.04 ± 0.57	11.13 ± 0.36	14.31 ± 0.19	18.16 ± 0.98
0.6	12.37 ± 0.78	17.57 ± 0.68	22.71 ± 0.99	28.58 ± 0.61
0.8	17.22 ± 0.82	23.45 ± 0.64	30.53 ± 0.98	37.94 ± 0.48
1.0	22.69 ± 0.87	29.91 ± 1.02	38.05 ± 0.86	46.50 ± 1.16
DMSO + water, *x*_SMT_ × 10^2^				
0.2	0.45 ± 0.02	0.81 ± 0.04	1.31 ± 0.04	1.89 ± 0.02
0.4	1.08 ± 0.05	2.41 ± 0.14	3.69 ± 0.15	5.22 ± 0.20
0.6	3.40 ± 0.16	4.98 ± 0.03	6.89 ± 0.25	9.32 ± 0.23
0.8	7.89 ± 0.34	10.16 ± 0.55	12.98 ± 0.69	16.54 ± 0.57
1.0	17.97 ± 0.66	21.05 ± 0.57	24.81 ± 0.10	29.30 ± 0.60
Acetonitrile + water, *x*_SMT_ × 10^3^				
0.2	3.24 ± 0.18	3.62 ± 0.10	4.03 ± 0.14	4.54 ± 0.17
0.4	9.02 ± 0.39	10.05 ± 0.23	11.22 ± 0.29	12.51 ± 0.38
0.6	12.04 ± 0.24	13.29 ± 0.36	14.62 ± 0.39	16.16 ± 0.18
0.8	6.92 ± 0.26	7.89 ± 0.37	8.91 ± 0.41	10.05 ± 0.43
1.0	2.83 ± 0.08	3.04 ± 0.07	3.21 ± 0.09	3.43 ± 0.08

## Data Availability

The data used in this paper are available on request from the corresponding author.

## Supplementary Materials

The following are available online at https://www.mdpi.com/article/10.3390/ma14205915/s1, Figure S1: Molar fraction solubility of Sulfamethizole in aqueous DMF binary solvents, Figure S2: Molar fraction solubility of Sulfamethizole in aqueous DMSO binary solvents, Figure S3: Molar fraction solubility of Sulfamethizole in aqueous acetonitrile binary solvents, Figure S4: Characteristics of solid Sulfamethizole residues obtained after shake-flask procedure, Figure S5: Distributions of the values of descriptors characterizing SMT in aqueous DMF binary mixtures at room temperature, Figure S6: Distributions of the values of descriptors characterizing SMT in aqueous DMSO binary mixtures at room temperature, Figure S7: Distributions of the values of descriptors characterizing SMT in aqueous 1,4-dioxane binary mixtures at room temperature, Figure S8: Distributions of the values of descriptors characterizing SMT in aqueous acetonitrile binary mixtures at room temperature, Figure S9: Distributions of the values of descriptors characterizing SMT in aqueous propylene glycol binary mixtures at room temperature, Table S1: List of SANN included in the ensemble of neural networks (ENN) for Sulfamethizole solubility prediction, Table S2: The environmental impact scores calculated using PARIS III (https://www.epa.gov/).
