# Supplementary material for: Experimental and Theoretical Screening for Green Solvents Improving Sulfamethizole Solubility
Source: Materials (Basel). 2021 Oct 9;14(20):5915. doi: 10.3390/ma14205915 (PMC8539550; doi:10.3390/ma14205915)
Supplement: Supplementary file 1 [file materials-14-05915-s001.zip › materials-1387417-supplementary.pdf]

Supplementary materials

Experimental and theoretical screening for green solvents improving sulfamethizole solubility

Piotr Cysewski [1,*] Maciej Przybyłek [1] and Rafal Rozalski [2]

[1] Department of Physical Chemistry, Faculty of Pharmacy, Collegium Medicum in Bydgoszcz, Nicolaus Copernicus University in Toruń, Kurpińskiego 5, 85-950 Bydgoszcz, Poland, m.przybylek@cm.umk.pl (M.P.)

[2] Department of Clinical Biochemistry, Faculty of Pharmacy, Collegium Medicum in Bydgoszcz, Nicolaus Copernicus University in Toruń, Karłowicza 24, 85-950 Bydgoszcz, Poland, rafalr@cm.umk.pl (R.R.);

* Correspondence: Piotr.Cysewski@cm.umk.pl

Table of contents

S.I. The results of solubility measurements performed for sulfamethizole in aqueous DMF, DMSO and acetonitrile 2

Figure S1. Molar fraction solubility of Sulfamethizole in aqueous DMF binary solvents. On the ordinate, x_2* represents the mole fractions of organic solvent in solute free binary solution. 2

Figure S2. Molar fraction solubility of Sulfamethizole in aqueous DMSO binary solvents. On the ordinate, x_2* represents the mole fractions of organic solvent in solute free binary solution. 2

Figure S3. Molar fraction solubility of Sulfamethizole in aqueous acetonitrile binary solvents. On the ordinate, x_2* represents the mole fractions of organic solvent in solute free binary solution. ... 3

S.II. The analysis of the solid sulfamethizole precipitate obtained after solubility determination procedure. 4

Figure S4. Characteristics of solid Sulfamethizole residues obtained after shake-flask procedure. .. 4

S.III. Descriptors distributions 5

Figure S5. Distributions of the values of descriptors characterizing SMT in aqueous DMF binary mixtures at room temperature. Series correspond to systems differing in mole fraction of organic solvent. 5

Figure S6. Distributions of the values of descriptors characterizing SMT in aqueous DMSO binary mixtures at room temperature. Series correspond to systems differing in mole fraction of organic solvent. 5

Figure S7. Distributions of the values of descriptors characterizing SMT in aqueous 1,4-dioxane binary mixtures at room temperature. Series correspond to systems differing in mole fraction of organic solvent. 6

Figure S8. Distributions of the values of descriptors characterizing SMT in aqueous acetonitrile binary mixtures at room temperature. Series correspond to systems differing in mole fraction of organic solvent. 6

Figure S9. Distributions of the values of descriptors characterizing SMT in aqueous propylene glycol binary mixtures at room temperature. Series correspond to systems differing in mole fraction of organic solvent. 7

S.IV. SANN details 8

Table S1. List of SANN included in the ensemble of neural networks (ENN) for Sulfamethizole solubility prediction. 8

S.V. Solvents environmental impact ranking 10

S.I. The results of solubility measurements performed for sulfamethizole in aqueous DMF, DMSO and acetonitrile

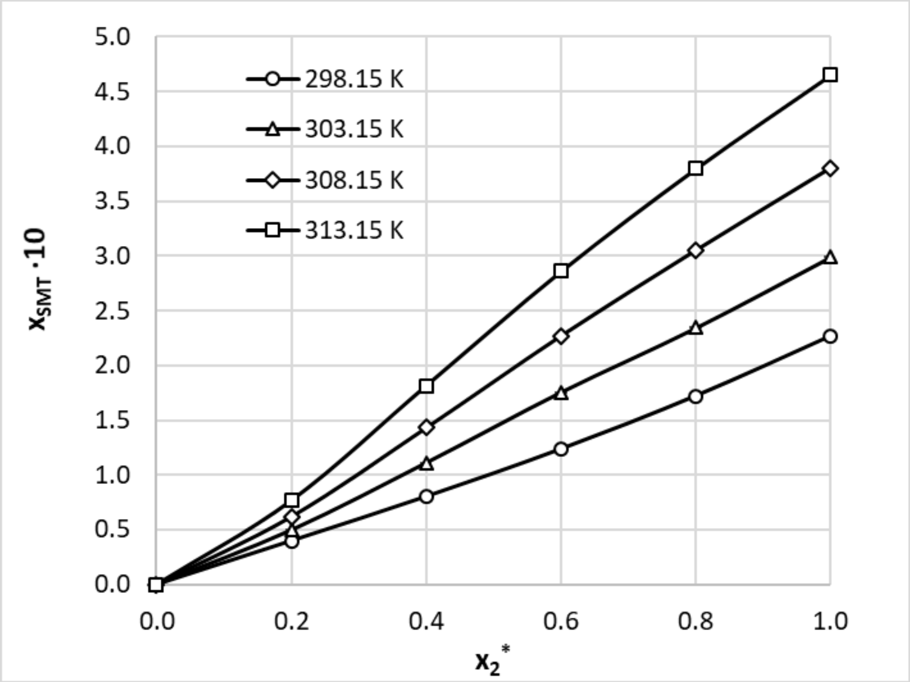

Figure S1. Molar fraction solubility of Sulfamethizole in aqueous DMF binary solvents. On the ordinate, x_2* represents the mole fractions of organic solvent in solute free binary solution.

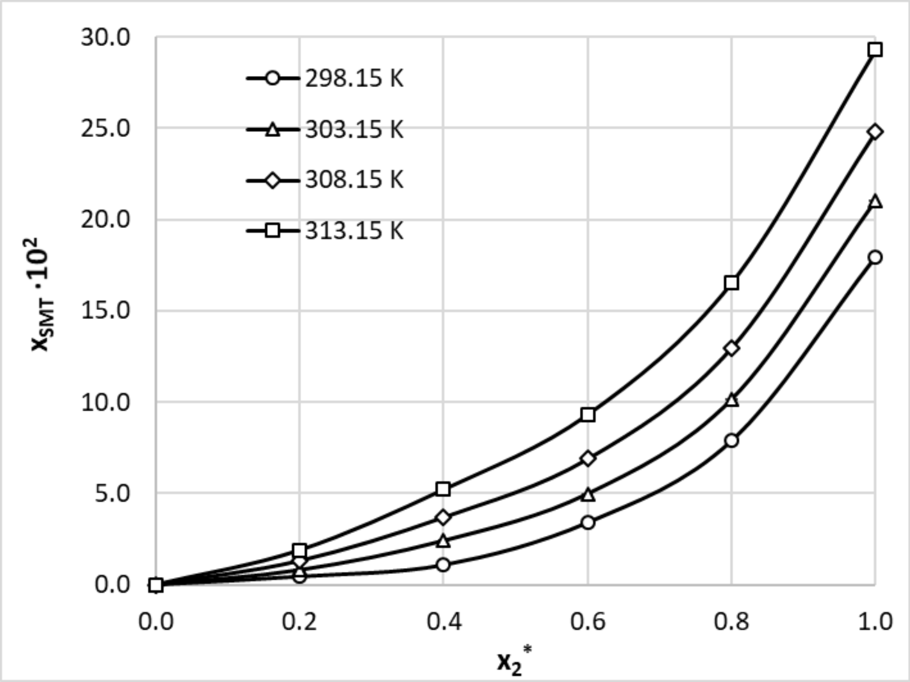

Figure S2. Molar fraction solubility of Sulfamethizole in aqueous DMSO binary solvents. On the ordinate, x_2* represents the mole fractions of organic solvent in solute free binary solution.

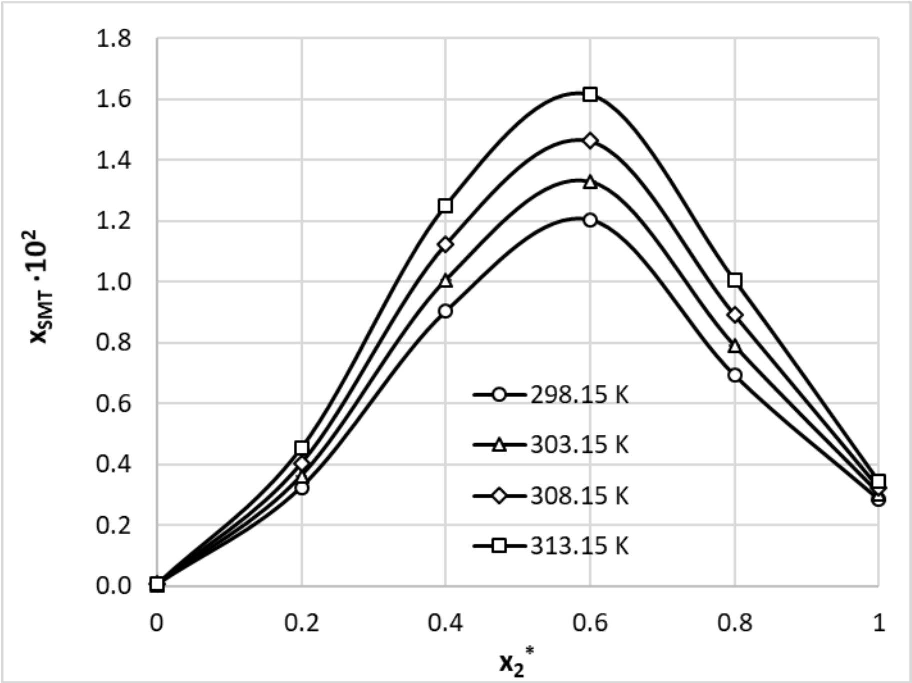

Figure S3. Molar fraction solubility of Sulfamethizole in aqueous acetonitrile binary solvents. On the ordinate, x_2* represents the mole fractions of organic solvent in solute free binary solution.

S.II. The analysis of the solid sulfamethizole precipitate obtained after solubility determination procedure.

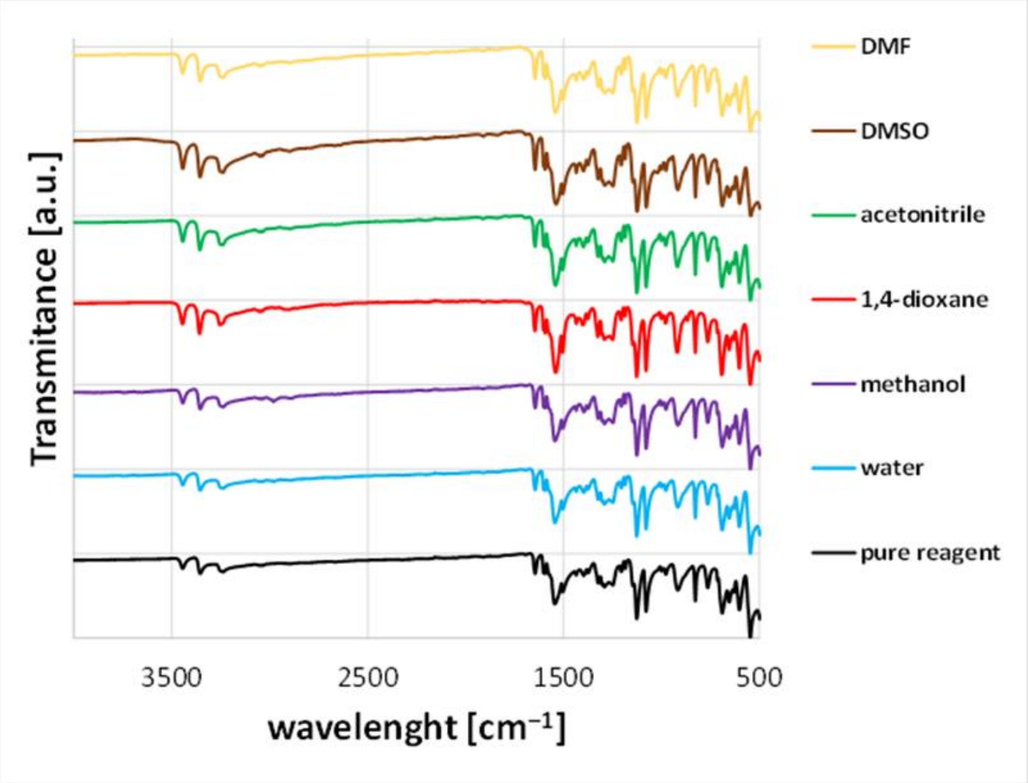

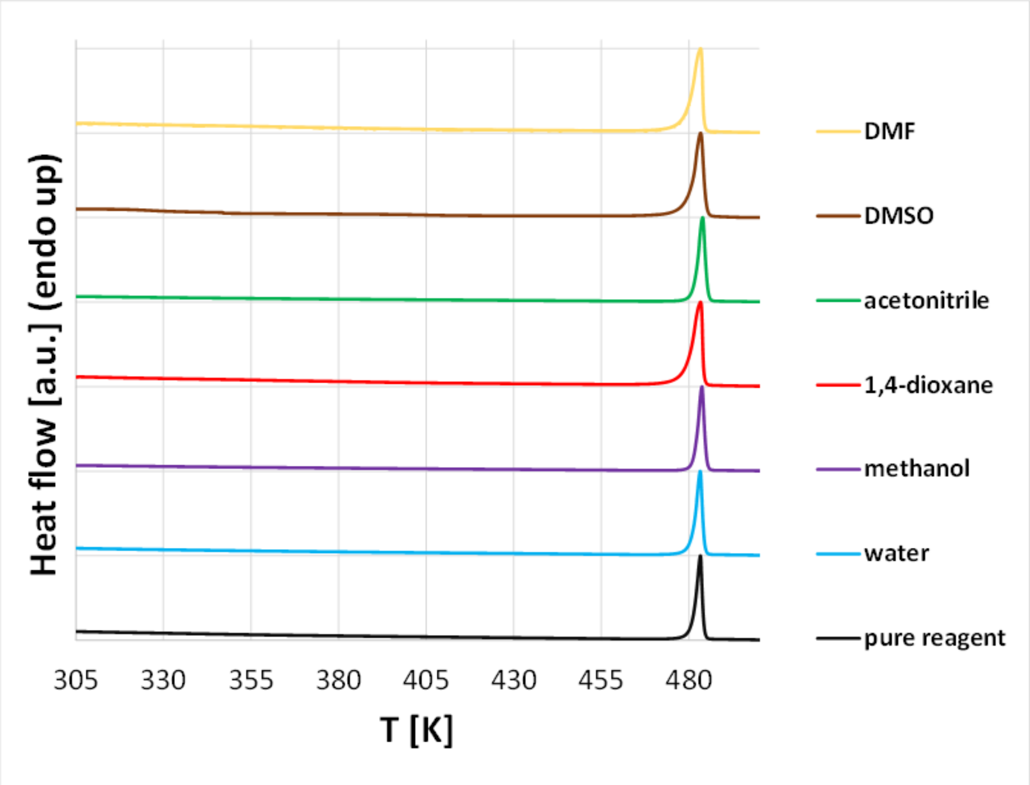

Figure S4. Characteristics of solid Sulfamethizole residues obtained after shake-flask procedure.

S.III. Descriptors distributions

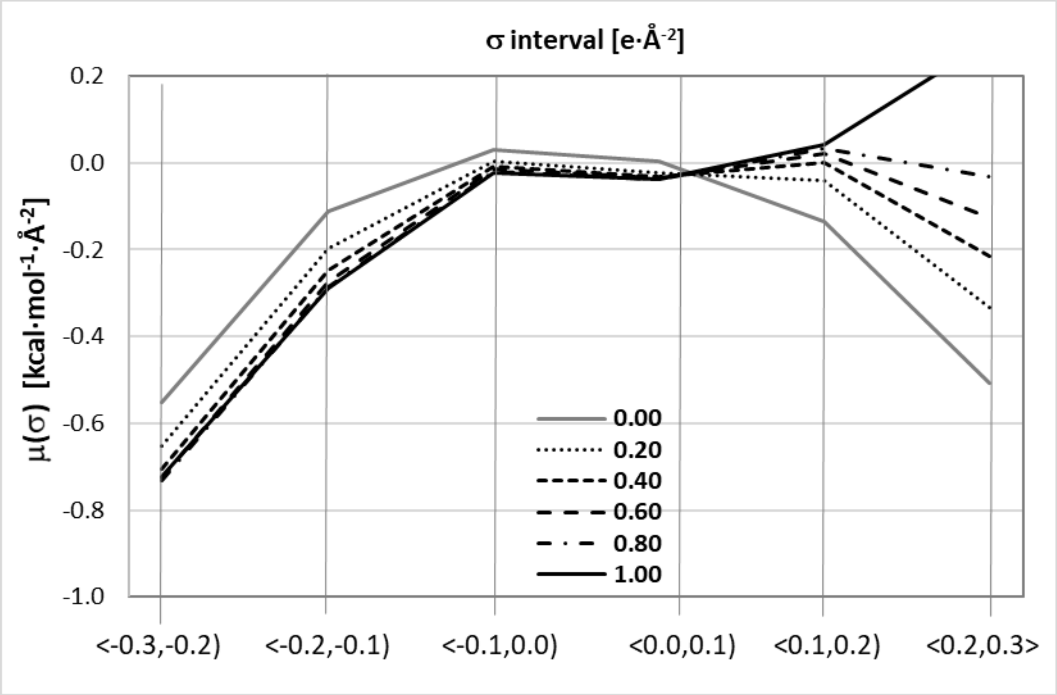

Figure S5. Distributions of the values of descriptors characterizing SMT in aqueous DMF binary mixtures at room temperature. Series correspond to systems differing in mole fraction of organic solvent.

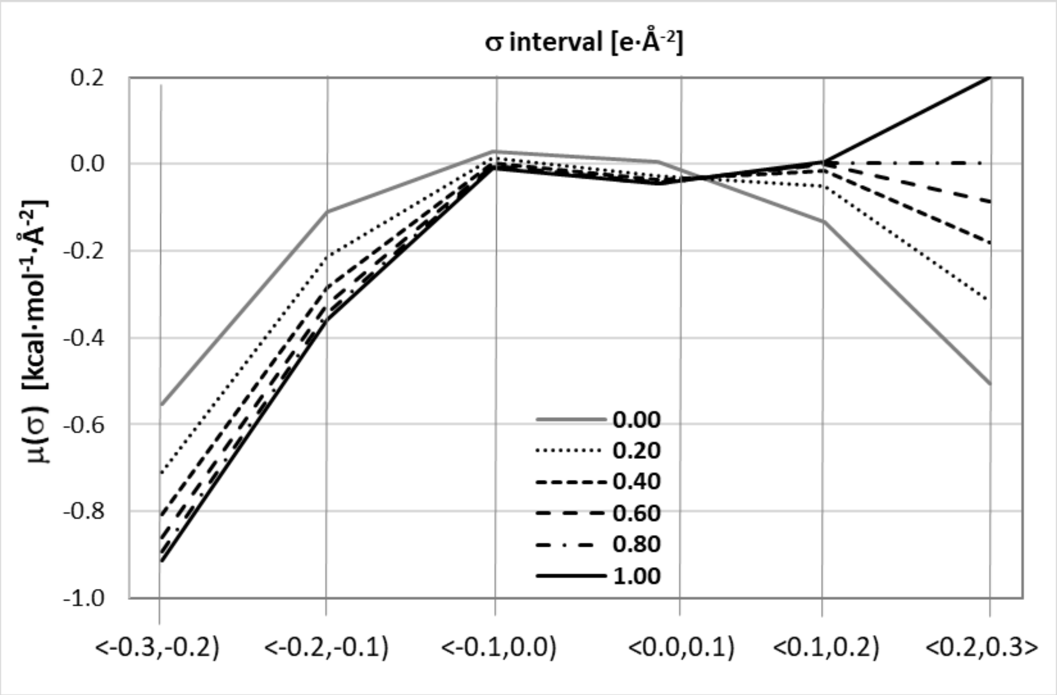

Figure S6. Distributions of the values of descriptors characterizing SMT in aqueous DMSO binary mixtures at room temperature. Series correspond to systems differing in mole fraction of organic solvent.

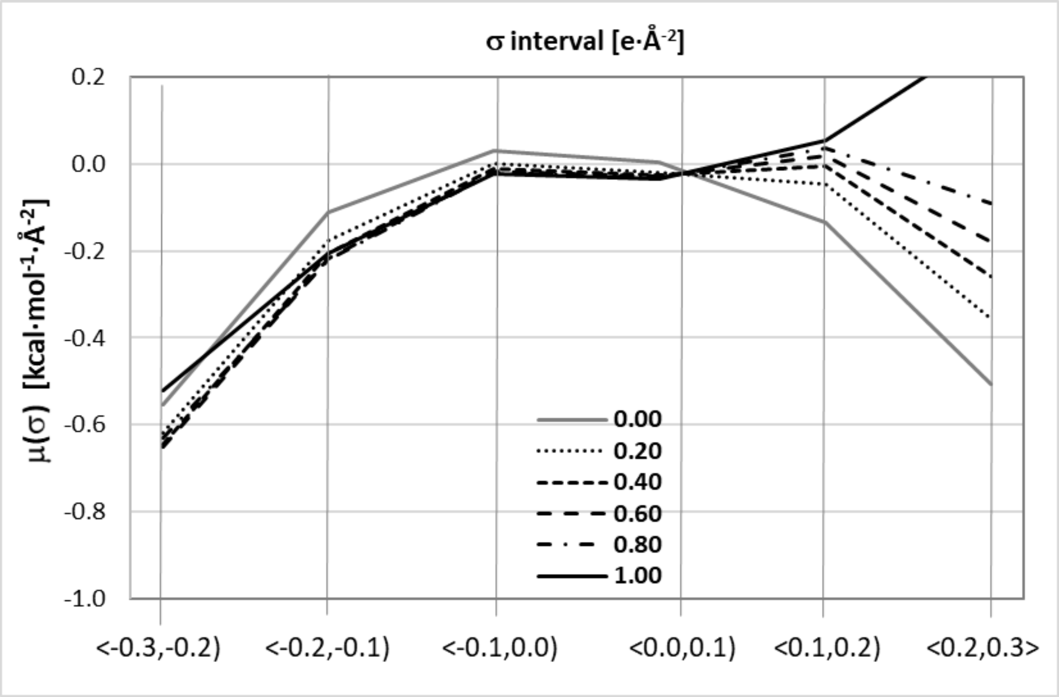

Figure S7. Distributions of the values of descriptors characterizing SMT in aqueous 1,4-dioxane binary mixtures at room temperature. Series correspond to systems differing in mole fraction of organic solvent.

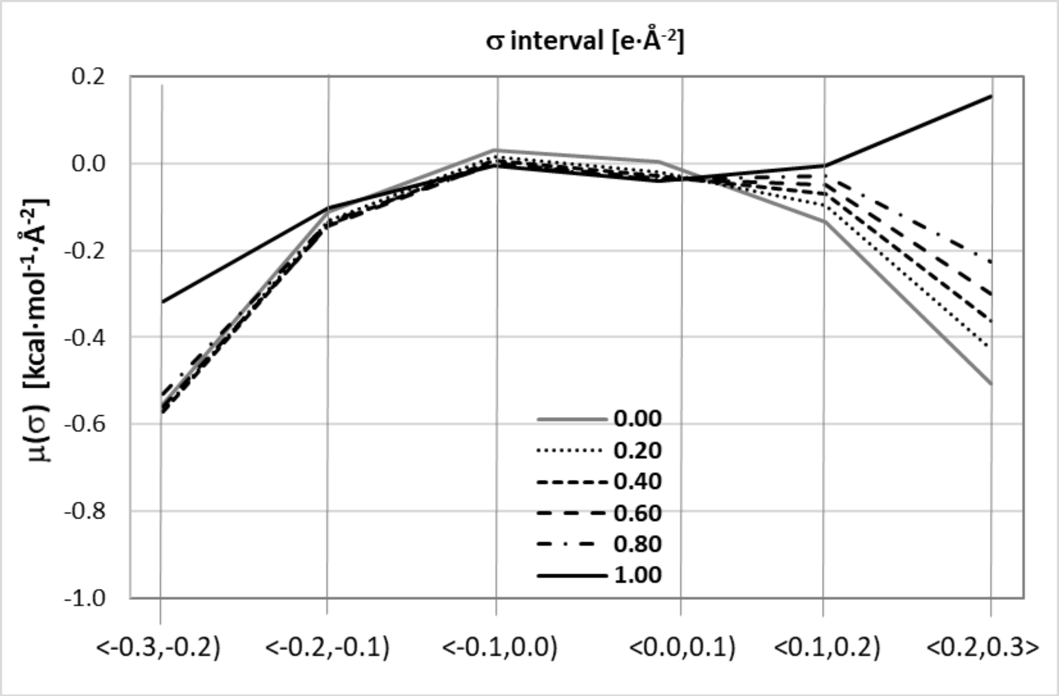

Figure S8. Distributions of the values of descriptors characterizing SMT in aqueous acetonitrile binary mixtures at room temperature. Series correspond to systems differing in mole fraction of organic solvent.

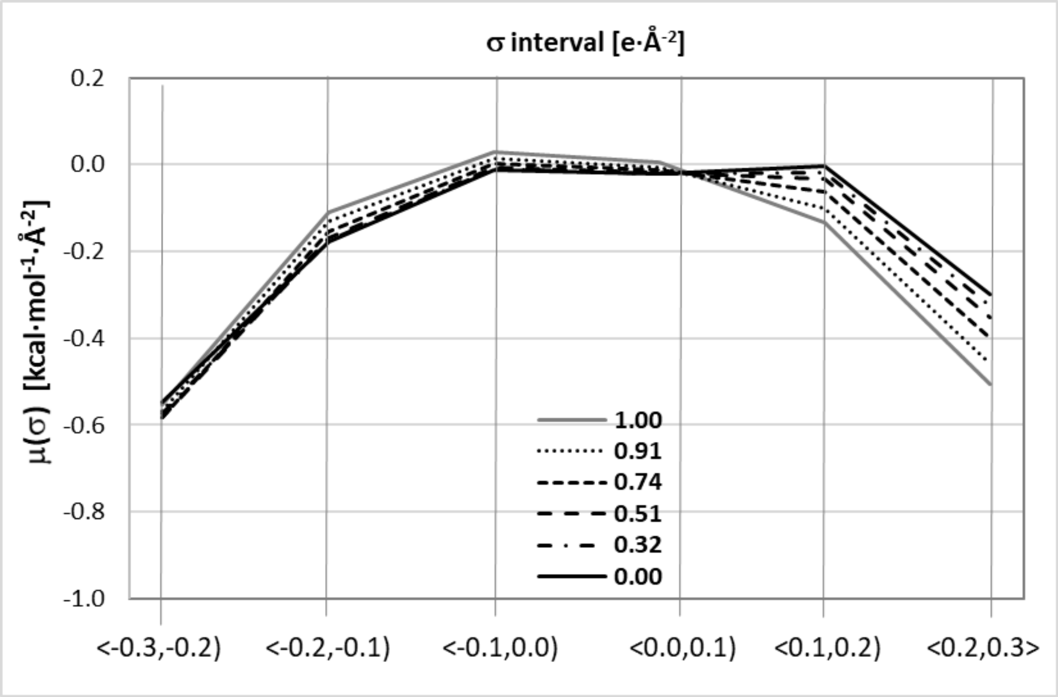

Figure S9. Distributions of the values of descriptors characterizing SMT in aqueous propylene glycol binary mixtures at room temperature. Series correspond to systems differing in mole fraction of organic solvent.

S.IV. SANN details

Table S1. List of SANN included in the ensemble of neural networks (ENN) for Sulfamethizole solubility prediction.

MLP	accuracy	precition	reliability	activation function	output function	algorithm
6-11-1	28.20	1	100%	Tanh	Logistic	BFGS 451
6-11-1	28.83	3	100%	Tanh	Tanh	BFGS 545
6-10-1	28.98	0	99%	Logistic	Linear	BFGS 423
6-10-1	29.08	1	97%	Tanh	Linear	BFGS 381
6-11-1	29.16	3	98%	Tanh	Linear	BFGS 423
6-11-1	29.34	3	99%	Tanh	Linear	BFGS 330
6-11-1	29.57	1	97%	Tanh	Linear	BFGS 307
6-11-1	29.78	2	95%	Tanh	Linear	BFGS 419
6-11-1	30.16	3	100%	Tanh	Logistic	BFGS 426
6-11-1	30.23	1	97%	Tanh	Linear	BFGS 277
6-10-1	30.32	1	97%	Tanh	Linear	BFGS 285
6-9-1	30.39	1	97%	Tanh	Linear	BFGS 332
6-9-1	30.60	2	99%	Tanh	Linear	BFGS 393
6-9-1	30.85	2	98%	Tanh	Linear	BFGS 503
6-8-1	30.92	3	97%	Tanh	Linear	BFGS 427
6-11-1	30.95	1	98%	Tanh	Linear	BFGS 479
6-11-1	31.00	1	97%	Tanh	Linear	BFGS 463
6-11-1	31.20	2	97%	Tanh	Exponential	BFGS 352
6-11-1	31.20	1	97%	Tanh	Linear	BFGS 540
6-11-1	31.25	1	97%	Tanh	Exponential	BFGS 282
6-11-1	31.27	2	98%	Tanh	Linear	BFGS 357
6-11-1	31.64	1	96%	Tanh	Linear	BFGS 326
6-9-1	32.05	0	97%	Tanh	Linear	BFGS 387
6-10-1	32.10	2	96%	Tanh	Exponential	BFGS 335
6-11-1	32.21	3	100%	Tanh	Logistic	BFGS 480
6-11-1	32.31	1	97%	Tanh	Linear	BFGS 242
6-11-1	32.32	2	97%	Tanh	Linear	BFGS 297
6-11-1	32.45	2	97%	Tanh	Linear	BFGS 375
6-9-1	32.55	3	97%	Tanh	Linear	BFGS 508
6-10-1	32.76	3	97%	Logistic	Linear	BFGS 408
6-9-1	32.80	3	98%	Tanh	Linear	BFGS 445
6-8-1	32.83	0	96%	Tanh	Linear	BFGS 439
6-10-1	33.18	3	98%	Tanh	Linear	BFGS 458
6-11-1	33.33	1	96%	Tanh	Exponential	BFGS 356
6-10-1	33.56	1	96%	Exponential	Linear	BFGS 517
6-11-1	33.62	2	100%	Tanh	Tanh	BFGS 421
6-7-1	33.77	3	96%	Tanh	Linear	BFGS 547
6-10-1	33.81	2	98%	Logistic	Linear	BFGS 505
6-8-1	33.82	2	97%	Tanh	Linear	BFGS 511
6-11-1	33.83	1	96%	Tanh	Linear	BFGS 308
6-11-1	33.83	2	97%	Logistic	Linear	BFGS 505

**MLP**	**accuracy**	**precition**	**reliability**	**activation function**	**output function**	**algorithm**
6-9-1	33.89	1	97%	Tanh	Linear	BFGS 368
6-10-1	34.13	0	96%	Tanh	Linear	BFGS 402
6-10-1	34.18	2	100%	Tanh	Logistic	BFGS 349
6-11-1	34.19	3	97%	Tanh	Linear	BFGS 356
6-10-1	34.25	2	100%	Tanh	Logistic	BFGS 365
6-10-1	34.38	1	96%	Logistic	Linear	BFGS 479
6-11-1	34.56	2	98%	Tanh	Linear	BFGS 248
6-10-1	34.65	2	97%	Tanh	Linear	BFGS 294
6-11-1	34.68	3	100%	Tanh	Logistic	BFGS 405
6-10-1	34.69	1	97%	Tanh	Linear	BFGS 371
6-11-1	34.74	2	100%	Tanh	Logistic	BFGS 336
6-11-1	34.78	3	96%	Tanh	Exponential	BFGS 306
6-11-1	34.83	2	97%	Tanh	Linear	BFGS 364
6-10-1	34.90	3	100%	Tanh	Tanh	BFGS 517
6-11-1	34.92	1	97%	Tanh	Linear	BFGS 354
6-11-1	34.96	3	100%	Tanh	Tanh	BFGS 479

S.V. Solvents environmental impact ranking

Table S2. The environmental impact scores calculated using PARIS III (https://www.epa.gov/). In all cases, the default program settings were used (impact factor for all environmental impact scores was 5). The following acronyms were used, HTPIng-Human Toxicity Potential by Ingestion, HTPInh -Human Toxicity Potential by Inhalation, TTP- Terrestrial Toxicity Potential, ATP- Aquatic Toxicity Potential, GWP- Global Warming Potential, ODP- Ozone Depletion Potential, PCOP-Photochemical Oxidation Potential, AR- Acid rain, EI-Environmental Index (Totals).

Solvent	CAS number	Environmental impact scores								EI[1]	Rank[1]
		HTPIng	HTPInh	TTP	ATP	GWP	OZP	PCOP	AR		
water	7732-18-5	0.010	0.000	0.010	0.000	0.000	0.000	0.000	0.000	0.020(0.020)	1(1)
4-formylmorpholine	4394-85-8	0.254	0.000	0.254	0.001	0.000	0.000	0.000	0.000	0.509(0.509)	2(4)
N-methylformamide	123-39-7	0.472	0.000	0.472	0.015	0.000	0.000	0.000	0.000	0.959(0.959)	3(8)
methanol-N-methylformamide (x_2*=0.2)	-	0.456	0.011	0.456	0.013	0.000	0.000	0.135	0.000	1.071(0.936)	4(7)
acetonitrile-water (x_2*=0.6)	-	0.596	0.267	0.596	0.002	0.000	0.000	0.000	0.000	1.461(1.461)	5(10)
DMF-N-methylformamide (x_2*=0.4)	-	0.564	0.364	0.564	0.008	0.000	0.000	0.000	0.000	1.500(1.500)	6(11)
acetonitrile	75-05-8	0.767	0.345	0.767	0.002	0.000	0.000	0.000	0.000	1.881(1.881)	7(13)
methanol	67-56-1	0.335	0.093	0.335	0.000	0.000	0.000	1.130	0.000	1.893(0.763)	8(5)
DMF	68-12-2	0.675	0.806	0.675	0.000	0.000	0.000	0.000	0.000	2.156(2.156)	9(14)
methanol-formamide (x_2*=0.4)	-	0.338	1.120	0.338	0.005	0.000	0.000	0.363	0.000	2.164(1.801)	10(12)
DMF-formamide (x_2*=0.8)	-	0.630	0.913	0.630	0.001	0.000	0.000	0.000	0.000	2.174(2.174)	11(15)
formamide	75-12-7	0.339	1.610	0.339	0.007	0.000	0.000	0.000	0.000	2.295(2.295)	12(16)
propylene glycol	57-55-6	0.094	0.000	0.094	0.000	0.000	0.000	4.310	0.000	4.499(0.189)	13(2)
1,4-dioxane-water (x_2*=0.6)	-	0.397	0.059	0.397	0.000	0.000	0.000	3.780	0.000	4.633(0.853)	14(6)
1,4-dioxane	123-91-1	0.450	0.067	0.450	0.000	0.000	0.000	4.300	0.000	5.267(0.967)	15(9)
DMSO	67-68-5	0.130	0.000	0.130	0.000	0.000	0.000	11.400	0.000	11.660(0.260)	16(3)

[1]In parenthesis the values of EI and ranks corresponds to ranking after exclusion of PCOP.
